# Alamandine alleviates hypertension and renal damage *via* oxidative-stress attenuation in Dahl rats

**DOI:** 10.1038/s41420-022-00822-y

**Published:** 2022-01-12

**Authors:** Juexiao Gong, Man Luo, Yonghong Yong, Shan Zhong, Peng Li

**Affiliations:** 1 Department of Cardiology, the Affiliated Hospital of Integrated Traditional Chinese and Western Medicine, Nanjing University of Chinese Medicine, Jiangsu Province Academy of Traditional Chinese Medicine, Nanjing, China; 2 Department of Emergency, The Affiliated Huaian No. 1 People’s Hospital of Nanjing Medical University, Huaian, China; 3 Department of Cardiology, the First Affiliated Hospital of Nanjing Medical University, Nanjing, China; 4 Department of Anesthesiology, Children’s Hospital of Nanjing Medical University, Nanjing, China

**Keywords:** Kidney diseases, Hypertension

## Abstract

Alamandine (Ala) is a novel member of the renin–angiotensin-system (RAS) family. The present study aimed to explore the effects of Ala on hypertension and renal damage of Dahl salt-sensitive (SS) rats high-salt diet-induced, and the mechanisms of Ala on renal-damage alleviation. Dahl rats were fed with high-salt diets to induce hypertension and renal damage in vivo, and HK-2 cells were treated with sodium chloride (NaCl) to induce renal injury in vitro. Ala administration alleviated the high-salt diet-induced hypertension, renal dysfunction, and renal fibrosis and apoptosis in Dahl SS rats. The HK-2 cells’ damage, and the increases in the levels of cleaved (c)-caspase3, c-caspase8, and c-poly(ADP-ribose) polymerase (PARP) induced by NaCl were inhibited by Ala. Ala attenuated the NaCl-induced oxidative stress in the kidney and HK-2 cells. DETC, an inhibitor of SOD, reversed the inhibitory effect of Ala on the apoptosis of HK-2 cells induced by NaCl. The NaCl-induced increase in the PKC level was suppressed by Ala in HK-2 cells. Notably, PKC overexpression reversed the moderating effects of Ala on the NaCl-induced apoptosis of HK-2 cells. These results show that Ala alleviates high-salt diet-induced hypertension and renal dysfunction. Ala attenuates the renal damage *via* inhibiting the PKC/reactive oxygen species (ROS) signaling pathway, thereby suppressing the apoptosis in renal tubular cells.

## Introduction

Hypertension is the most prevalent cardiovascular disease and the major risk factor for the high morbidity and mortality of chronic disease, which have caused overwhelming global economic and health problems [1]. High-salt intake is a risk factor for hypertension, stroke, and cardiovascular diseases [2]. A relationship exists between dietary salt intake and hypertension [3]. Although the positive correlation relationship between dietary salt intake and high blood pressure has been well described, the precise mechanisms of salt-induced hypertension are still poorly understood.

Refractory hypertension is common in chronic kidney-disease patients and conveys an increased risk for adverse cardiovascular outcomes and the development of renal failure [4]. The link between inappropriate salt retention in the kidney and hypertension is well known. The pathogenesis of salt-induced kidney-associated hypertension is not completely understood, but an imbalance of sodium and chloride homeostasis seems to be involved in both the induction and the development of salt-sensitive hypertension [5]. Sodium chloride (NaCl) diets were found to cause interstitial fibrosis, tubular dilatation, glomerular sclerosis, and tubular epithelial-cell apoptosis in rats [6, 7].

Alamandine (Ala) is a new component of the renin–angiotensin system (RAS), which can be formed by either the decarboxylation of angiotensin (Ang)-(1–7) or the hydrolysis of Ang A [8]. RAS is not only an important physiological regulator of the heart and kidney homeostasis, but also plays pivotal roles in the pathophysiology of heart and kidney diseases. Although Ala can alleviate essential hypertension [9], it is still not well known whether it can attenuate salt intake-induced high blood pressure. Ala alleviates cardiovascular diseases by oxidative-stress suppression [10, 11]. An earlier study showed that Ala prevented doxorubicin-induced nephrotoxicity induced in rats [12]. Furthermore, Ala is a fundamental inhibitor of oxidative stress, which is critically involved in renal dysfunction and hypertension [13–15]. Thus, we hypothesized that Ala can alleviate hypertension and renal damage *via* oxidative-stress attenuation. Therefore, in the current study, we explored whether Ala attenuated high-salt-induced hypertension and renal dysfunction in Dahl salt-sensitive (SS) rats. We also sought to examine the potential roles of Ala in NaCl-induced HK-2 cell damage, as well as the associated underlying mechanisms.

## Materials and methods

### Animals and Ala treatment

About 4–5-week-old male Dahl salt-sensitive (SS) and Dahl salt-resistant (SR) rats (Vital River Biological Co., Ltd, Beijing, China) were housed in a temperature-controlled room on a 12–12 h light–dark cycle with *ad libitum* access to standard chow and tap water. All procedures were approved by the Experimental Animal Care and Use Committee of Nanjing Medical University and conducted in compliance with the Guide for the Care and Use of Laboratory Animals (NIH publication No. 85-23, revised 1996). The rats were fed a control diet (0.4% NaCl) for 10 days and HSD (4% NaCl; Research Diets Inc., NJ, USA) for 30 days. All the rats were randomly equally divided into saline or Ala-treated groups. Ala (50, 500, or 5000 µg/kg/d, Phoenix Pharmaceuticals Inc., CA, USA) or the same volume of saline was administered by intraperitoneal injection for 20 days (8 rats per group).

### Blood-pressure measurements

Rats were implanted with telemetry devices (Data Sciences International Inc., MN, USA) through the abdominal aorta as described previously [16]. More specifically, rats were paced on a heated surgical field in dorsal recumbency under anesthesia with isoflurane. A midline incision in the abdomen was made, and the abdominal aorta was isolated. The rat was implanted with a telemetry device, with a catheter inserted into the abdominal aorta and the transmitter body implanted intraperitoneally. Arterial blood pressure was remotely monitored using a commercially available radiotelemetry data-acquisition program (Ponemah v6.1, Data Sciences International Inc., MN, USA).

### Biochemical assays of Cr, BUN, and CysC

After 30-day HSD, blood samples were collected and centrifuged at 3000 g and 4 °C for 10 min. An automated analyzer (AU 5800, Beckman Coulter, Indianapolis, IN, USA) was used to measure the levels of serum creatinine (Cr) and blood urea nitrogen (BUN). The level of serum cystatin C (CysC) was analyzed by an ELISA kit (Elabscience Biotechnology Co., Ltd., Wuhan, China).

### Masson staining

Kidney sections (5 µm) were stained with Masson staining (Service Biological Technology Co., Ltd, Wuhan, China) to evaluate renal fibrosis. The sections were obtained under a light microscope (Carl Zeiss, Oberkochen, Germany), and analyzed by image-Pro Plus software (Media Cybernetics, Inc., MD, USA).

### Terminal deoxyribonucleotidyl transferase (TdT)-labeling assay

The renal samples were then fixed with 4% paraformaldehyde, embedded in paraffin, and sectioned into 5-cm-thick slides. Apoptosis was determined by terminal deoxyribonucleotidyl transferase (TdT)-labeling (TUNEL) assay using an in situ cell death detection kit (Roche Diagnostics, Mannheim, Germany) following the kit instructions. Images were obtained using a fluorescence microscope (Carl Zeiss) under 200× magnification.

### Immunofluorescence

The renal samples were then fixed with 4% paraformaldehyde, embedded in paraffin, and sectioned into 5-cm-thick slides. Then, the samples were incubated with primary antibody against Bax (#14796; Cell Signaling Technology, MA, USA), c-caspase3 (#9661; Cell Signaling Technology), and 8-hydroxy-2′ -deoxyguanosine (8-OHdG; #sc-66036; Santa, TX, USA) at 4 °C overnight, followed by the corresponding secondary antibodies (Jackson ImmunoResearch, PA, USA) for 2 h at room temperature. Then, 4′,6-diamidino-2-phenylindole (DAPI; Life Technologies Co., NY, USA) was used to counterstain the nucleus. The images were captured using a fluorescence microscope (Carl Zeiss GmbH, Oberkochen, Germany).

### Cell culture and treatment

HK-2 cells were purchased from the Chinese Academy of Sciences (Shanghai, China) and cultured in Dulbecco’s modified Eagle’s medium (DMEM; Invitrogen Life Technologies, CA, USA), supplemented with 10% fetal bovine serum (Gibco, Shanghai, China), 100 U/mL penicillin, and 100 mg/mL streptomycin (Invitrogen Life Technologies) at 37 °C in a humidified, 5% CO_2_, 95% air atmosphere. HK-2 cells were treated with NaCl (50 mM) for 24 h. Ala (0.1, 1.0, and 10 ug/mL) was added to the NaCl + Ala group simultaneously with the addition of NaCl. Recombinant adenoviral vectors harboring PKC (Ad-PKC; GeneChem, Shanghai, China) were concurrently added to the NaCl + Ala + Ad-PKC and NaCl + Ala cultures.

### Quantitative real-time PCR

Total RNA was extracted from the renal samples using TRIzol (Ambion, TX, USA). cDNA was obtained by reverse transcription with random primers (GenScript, Nanjing, China) following the instructions of the PrimeScript™ RT Master Mix (TaKaRa, Beijing, China), and was then stored at −80 °C until use. SYBR Green I fluorescence was utilized for mRNA determination. All samples were placed in a 384-well plate and amplified in triplicates in 35 cycles. The relative gene expression was next calculated using the values of the Δcycle threshold (ΔCt) as a relative quantity for endogenous control. The primers used for qRT-PCR are shown in Table 1.

**Table 1:** List of utilized primers for qRT-PCR.

Gene	Species	Forward primer	Reverse primer
NGAL	Human	TCACCTCCGTCCTGTTTAG	CTCCTTGGTTCTCCCGTA
KIM-1	Human	CTGCAGGGAGCAATAAGGAG	ACCCAAAAGAGCAAGAAGCA
GAPDH	Human	CCACATCGCTCAGACACCAT	CCAGGCGCCCAATACG

### Western blotting

Protein levels were determined using Western blot. Briefly, cells or tissues were sonicated in RIPA lysis buffer and homogenized. Cell debris was removed, and the supernatant was obtained by centrifugation for 10 min at 12,000 *g* and 4 °C. After electrophoretic separation and transfer of bands to a polyvinyl difluoride (PVDF) membrane, the proteins on the membrane were probed with antibodies against c-caspase3 (#9661; Cell Signaling Technology), c-caspase8 (#9496; Cell Signaling Technology), c-PRAP (#5625; Cell Signaling Technology), Rac1 (#4651; Cell Signaling Technology), p22^phox^ (ab80896; Abcam, Shanghai, China), p47^phox^ (ab181090; Abcam), NOX4 (ab133303; Abcam), and PKC (#2056; Cell Signaling Technology). Glyceraldehyde phosphate dehydrogenase (GAPDH; ab181602; Abcam) was used as loading controls. The total protein level was normalized to the GAPDH protein level.

### Determination of the superoxide dismutase activity

Renal tissue and HK-2 cells were obtained and homogenized in lysis buffer (Thermo Fisher Scientific, MA, USA). The superoxide dismutase (SOD) activity in the renal tissues was measured following the manufacturer’s instructions (Jiancheng Bioengineering Institute, Nanjing, China).

### Determination of glutathione and malondialdehyde levels

Renal tissue and HK-2 cells were then homogenized in lysis buffer (Thermo Fisher Scientific). The malondialdehyde (GSH) and malondialdehyde (MDA) levels were determined by ELISA kits (USCN Business Co., Ltd., Wuhan, China) following the manufacturer’s instructions.

### Measurement of superoxide anions

Next, the superoxide anion level in the renal tissue and HK-2 cells was detected by lucigenin-derived chemiluminescence. Briefly, dark-adapted lucigenin (5 μM) was added to each supernatant sample to produce photon emission. The photon emission was detected using a microplate reader (BioTek) once every minute for 10 min. The values representing the superoxide anion level were expressed as the MLU per minute per milligram of protein.

### Statistical analyses

Data were expressed as mean ± SEM. Sample size for each experiment was calculated by analyzing preliminary–experimental data with the PASS statistical software (UT, USA). The statistically significant differences among the groups were blindly assessed by one-way analysis of variance (ANOVA) using the Bonferroni’s post hoc test with GraphPad Prism (Version 7.0; CA, USA). Two-tailed *P*-values < 0.05 were considered to indicate statistical significance.

## Results

### Ala alleviated high-salt diet-induced renal dysfunction and hypertension of Dahl SS rats

**Fig. 1:**
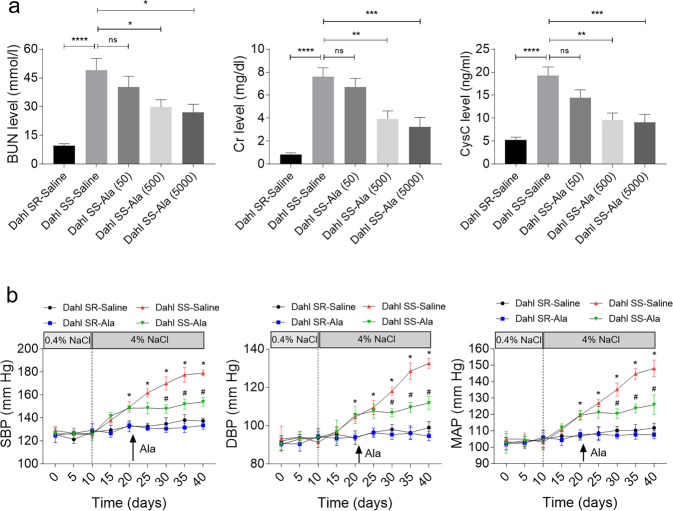
Alamandine (Ala) alleviated renal dysfunction and hypertension of Dahl salt-sensitive (SS) rats induced by high-salt diet. **a** Ala (500 and 5000 μg/kg/d) inhibited the increases in BUN, Cr, and CysC in Dahl SS rats; (**b**) Ala (500 μg/kg/d) inhibited the increases in the systolic blood pressure (SBP), diastolic blood pressure (DBP), and mean arterial pressure (MAP) in Dahl SS rats. The data are expressed as mean ± standard error of the mean (SEM); *n* = 8 in each group. *P < 0.05, **P < 0.01, ***P < 0.001, and ****P < 0.0001; #*P* < 0.05 vs. Dahl SS-saline group.

The levels of BUN, Cr, and CysC were higher in Dahl SS rats than in Dahl SR rats that received high-salt diet. The increases of BUN, Cr, and CysC were inhibited by Ala administration in Dahl SS rats. The medium dose (500 μg/kg/d) or high dose (5000 μg/kg/d), but not the low dose (50 μg/kg/d) of Ala attenuated the increases in BUN, Cr, and CysC in the Dahl SS rats subjected to high-salt diet. The high dose of Ala did not cause further suppressive effects on the BUN, Cr, and CysC levels as compared with those of the medium dose of Ala (Fig. 1a). Therefore, the medium dose (500 μg/kg/d) was selected in the subsequent in vivo studies. The high-salt diet increased the levels of SBP, DBP, and MAP in Dahl SS rats, but such effects were not observed in Dahl SR rats. Ala administration blocked the high-salt diet-induced increases in SBP, DBP, and MAP in Dahl SS rats (Fig. 1b).

### Ala alleviated the renal damage of Dahl SS rats induced by high-salt diet

**Fig. 2:**
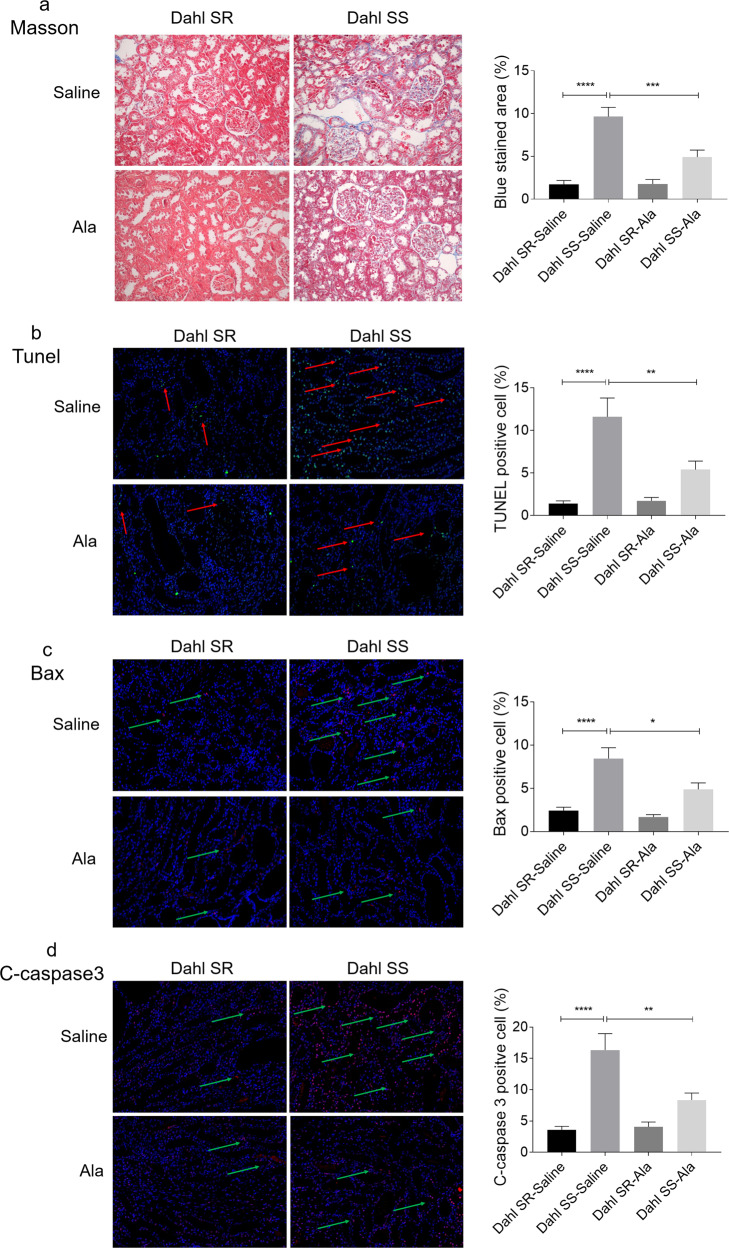
Alamandine (Ala) alleviated renal damage of Dahl salt-sensitive (SS) rats induced by high-salt diet. **a** The increased renal fibrosis in Dahl SS rats induced by high-salt diet was inhibited by Ala (200 X). **b** The increase of the TUNEL-positive cell number in the kidney of Dahl SS rats induced by high-salt diet was suppressed by Ala (200 X). **c** The increase of the Bax-positive cell number in the kidney of Dahl SS rats induced by high-salt diet was suppressed by Ala (200 X). **d** The increase of the c-caspase3-positive cell number in the kidney of Dahl SS rats induced by high-salt diet was suppressed by Ala (200 X). The data are expressed as mean ± standard error of the mean (SEM); *n* = 8 in each group. *P < 0.05, **P < 0.01, ***P < 0.001, and ****P < 0.0001; #*P* < 0.05 vs. Dahl SS-saline group.

The high-salt diet promoted the fibrosis of the kidney in Dahl SS rats, but this increase was effectively inhibited by the administration of Ala (Fig. 2a). The number of TUNEL-positive cells in the kidney of Dahl SS rats fed with high-salt diet was higher than that in Dahl SR rats, which was reversed by Ala treatment (Fig. 2b). The numbers of Bax (Fig. 2c) and c-cleaved 3- (Fig. 2d) positive cells were increased in the kidney of Dahl SS rats, and these increases were attenuated by Ala administration.

### Ala alleviated the renal tubular cell damage induced by NaCl

**Fig. 3:**
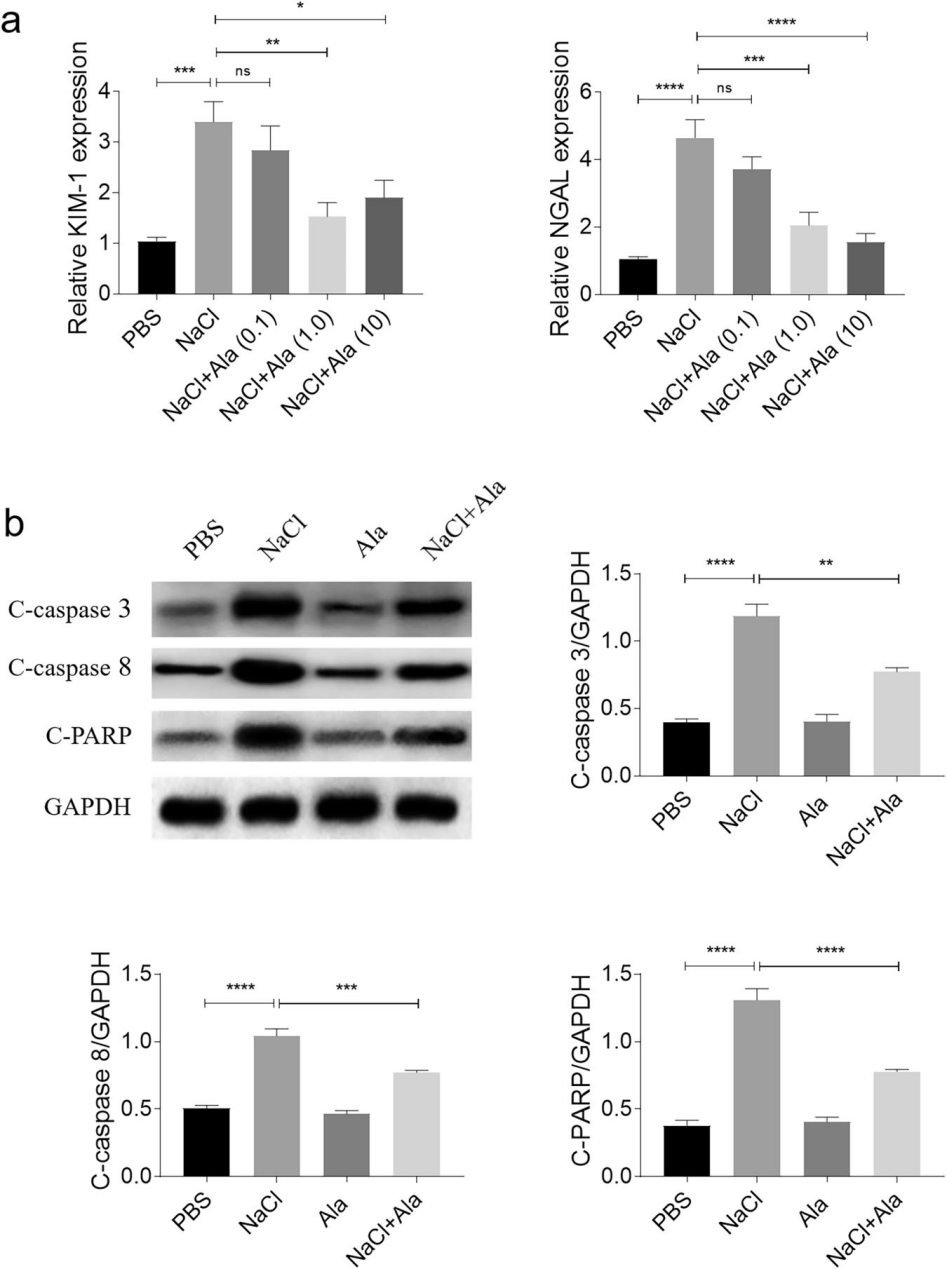
Alamandine (Ala) alleviated renal tubular cell damage induced by sodium chloride (NaCl). **a** Ala (1.0 and 10 µg/mL) inhibited the increases of KIM-1 and NGAL in HK-2 cells induced by NaCl. **b** Ala (1.0 µg/mL) inhibited the increases in cleaved (**c**)-caspase3, c-caspase8, and c-poly(ADP-ribose) polymerase (PARP) in HK-2 cells induced by NaCl. The data are expressed as mean ± standard error of the mean (SEM); *n* = 8 (**a**) or 4 (**b**) in each group. *P < 0.05, **P < 0.01, ***P < 0.001, and ****P < 0.0001.

The expression levels of KIM-1 and NGAL were augmented in the HK-2 cells treated with NaCl. The elevation in the KIM-1 and NGAL levels induced by NaCl was suppressed by Ala administration in the HK-2 cells. The medium dose (1.0 µg/mL) or the high dose (10 μg/mL), but not the low dose (0.1 µg/mL) of Ala blocked the NaCl-induced rise in KIM-1 and NGAL in the HK-2 cells. Notably, the high dose of Ala did not promote more pronounced attenuating effects on the KIM-1 and NGAL levels than the medium dose of Ala (Fig. 3a). Therefore, the medium dose (1.0 µg/ml) was selected for the subsequent in vitro studies. The levels of c-caspase3, c-caspase8, and c-PARP were elevated in the HK-2 cells treated with NaCl, but the Ala treatment suppressed that rise (Fig. 3b).

### Ala alleviated the renal tubular cell oxidative stress induced by NaCl

**Fig. 4:**
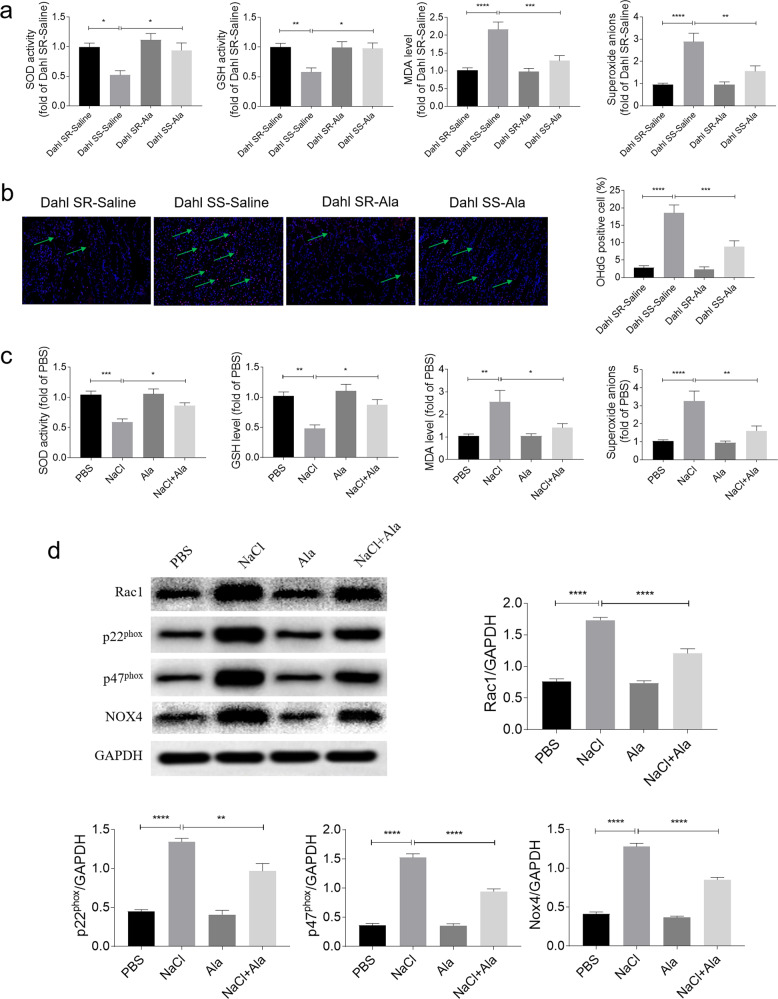
Alamandine (Ala) alleviated renal oxidative stress induced by sodium chloride (NaCl). **a** Ala reversed the decreases of superoxide dismutase (SOD) activity and glutathione peroxidase (GSH) level, and the increases in malonaldehyde (MDA) and superoxide anions in the kidney of Dahl salt-sensitive (SS) rats induced by high-salt diet. **b** The increase of the 8-OHdG-positive cell number in the kidney of Dahl SS rats induced by high-salt diet was suppressed by Ala (200 X). **c** Ala reversed the decreases of SOD activity and GSH level, and the increases in MDA and superoxide anions in HK-2 cells induced by NaCl. **d** Ala inhibited the rise in glutathione S-transferase (GST)—Ras-related C3 botulinum-toxin substrate 1 (Rac1), p22^phox^, p47^phox^, and NADPH oxidase 4 (NOX4) in HK-2 cells induced by NaCl. The data are expressed as mean ± standard error of the mean (SEM); *n* = 8 (**a**) or 4 (**b**) in each group. *P < 0.05, **P < 0.01, ***P < 0.001, and ****P < 0.0001.

The levels of the SOD activity and GSH level in the kidney of Dahl SS rats were reduced, but these decreases were reversed by administration of Ala. The MDA and superoxide anion levels were elevated in the kidney of Dahl SS rats, and these increases were attenuation by Ala administration (Fig. 4a). 8-OHdG is one of the predominant forms of free radical-induced oxidative lesions, and has therefore been widely used as a biomarker for oxidative stress. The number of 8-OHdG-positive cells in the kidney of Dahl SS rats was higher than that in Dahl SR rats, which was reversed by treating with Ala (Fig. 4b). The levels of the SOD activity and GSH level in the HK-2 cells treated with NaCl were reduced, but these decreases were reversed by the Ala treatment. The MDA and superoxide anion levels were elevated in the NaCl-treated HK-2 cells; however these increases were also suppressed by treatment with Ala (Fig. 4c). The levels of GST-Rac1, p22^phox^, p47^phox^, and NOX4 were increased in the HK-2 cells treated with NaCl, but Ala had an inhibitory influence on this effect (Fig. 4d).

### Oxidative-stress enhancement reversed the alleviating effects of Ala on the renal tubular cell damage induced by NaCl

**Fig. 5:**
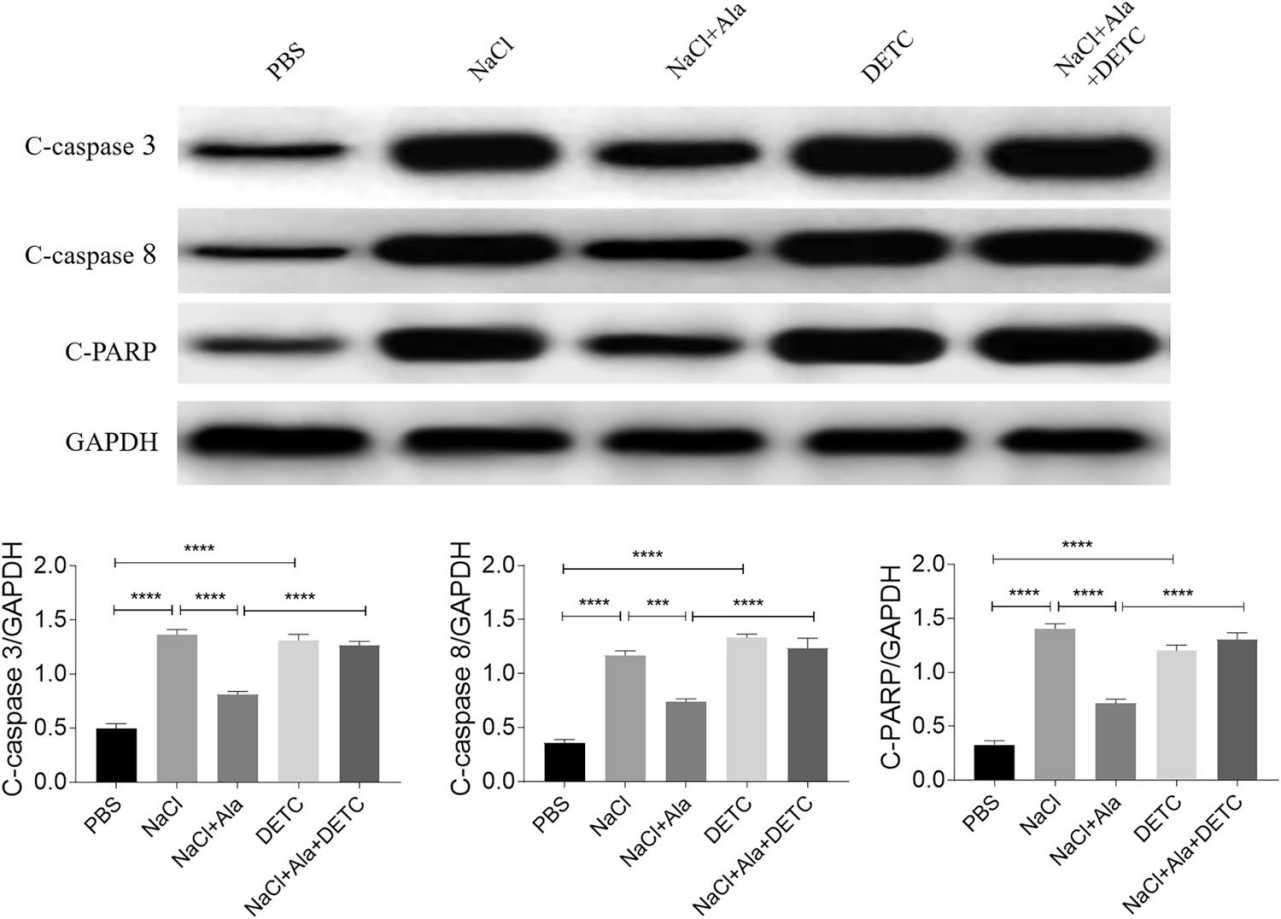
Oxidative-stress enhancement reversed the alleviating effects of alamandine (Ala) on renal tubular cell damage induced by sodium chloride (NaCl). Diethyldithiocarbamate (DETC) reversed the inhibitory effect of Ala on the increase in cleaved (c)-caspase-3, c-caspase-8, and c-poly(ADP-ribose) polymerase (PARP) induced by NaCl in HK-2 cells. The data are expressed as mean ± standard error of the mean (SEM); *n* = 4 in each group. *P < 0.05, **P < 0.01, ***P < 0.001, and ****P < 0.0001.

DETC, an inhibitor of SOD, reversed the inhibitory effect of Ala on the increase of c-caspase-3 induced by NaCl in HK-2 cells. The attenuating effect of Ala on the increase of c-caspase-8 induced by NaCl was reversed by SOD inhibitor DETC. In addition, DETC reversed the alleviating effect of Ala on the increase of c-PARP induced by NaCl in HK-2 cells (Fig. 5).

### The PKC signaling pathway is involved in the effects of Ala

**Fig. 6:**
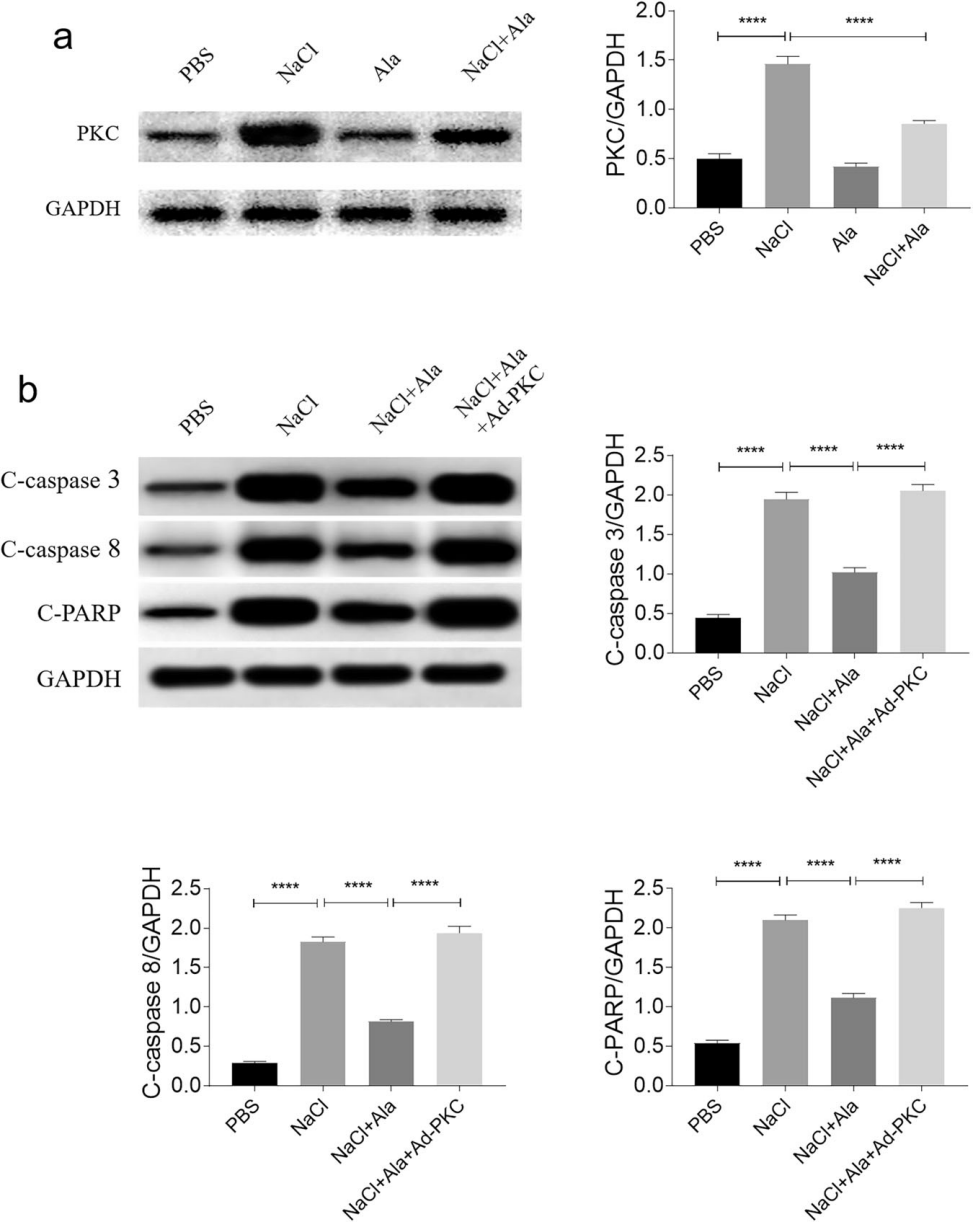
PKC signaling pathway was involved in the effects of alamandine (Ala) on attenuating apoptosis of renal tubular cell damage induced by sodium chloride (NaCl). **a** Ala inhibited the increase of PKC in the HK-2 cells induced by NaCl. **b** PKC overexpression reversed the attenuating effects of Ala on the increases of cleaved (c)-caspase3, c-caspase8, and c-poly(ADP-ribose) polymerase (PARP) induced by NaCl in HK-2 cells. The data are expressed as mean ± standard error of the mean (SEM); *n* = 4 in each group. *P < 0.05, **P < 0.01, ***P < 0.001, and ****P < 0.0001.

The PKC level was increased in the NaCl-treated HK-2 cells, but this rise was inhibited by the Ala treatment (Fig. 6a). The inhibitory effect of Ala on the increase of c-caspase3 induced by NaCl in the HK-2 cells was reversed by PKC overexpression. In addition, the attenuating effects of Ala on the increases of c-caspase8, and c-PARP induced by NaCl in the HK-2 cells, were also reversed by PKC overexpression (Fig. 6b).

## Discussion

The novel findings of the present study are that Ala alleviated the high-salt intake-induced hypertension and renal damage in Dahl rats. The high-salt diet-induced kidney fibrosis in Dahl rats was attenuated by Ala administration. Moreover, the NaCl-induced apoptosis of the tubular epithelial cells was reduced by treating with Ala. Ala inhibited the apoptosis of tubular epithelial cells induced by NaCl via attenuating oxidative stress. PKC signaling pathway was involved in the suppressive effect of Ala on the increase of tubular epithelial cells’ apoptosis induced by NaCl.

Ala is similar to Ang-(1–7); it differs only by the presence of an alanine residue in place of an aspartate residue at the amino end [8]. In previous study, Ala attenuated essential [9] and renovascular hypertension [17]. However, whether Ala can alleviate high-salt intake-associated hypertension has not been well elaborated. We found that the administration of Ala alleviated the SBP, DBP, and MAP levels of Dahl rats fed with high-salt diets. This result indicates that Ala can alleviate high-salt-induced hypertension.

Populations with a specific subtype of hypertension, salt-sensitive hypertension, manifested by significant changes in the blood pressure in response to salt intake, are at a higher risk for renal disease [18]. High-salt diet is considered a public health concern worldwide, which is associated with renal diseases [19]. Ala was reported to improve renal dysfunction of ischemia and reperfusion rats [20]. The Dahl SS rat is a well-established and characterized rat model of salt-sensitive hypertension, which displays renal lesions virtually identical to those in human hypertensive nephrosclerosis [21]. In the present study, we found that Ala treatment suppressed the high-salt-induced increases in the levels of BUN, Cr, and CysC in Dahl rats. The rise in KIM-1 and NGAL in HK-2 cells induced by NaCl was inhibited by Ala treatment. In addition, high salt caused kidney fibrosis in the Dahl SS rats, which was improved by Ala administration. These results indicate that Ala can alleviate the renal dysfunction induced by high-salt intake.

High salt has been found to cause apoptosis in organs, including the heart [22], brain [23], and the kidney [24]. Organ-specific cell death or apoptosis involving both parenchymal and microvasculature endothelial cells is conceivably underlying organ dysfunction [25]. In a previous study, Ala inhibited the increase in the apoptosis in the heart of sepsis mice [26]. However, whether Ala can attenuate the apoptosis of the kidneys induced by high salt is not well elucidated. The results of this study showed that the high-salt diet-induced increase in the quantities of TUNEL, Bax, and c-caspase3-positive cells in the kidneys of Dahl rats was attenuated by Ala. In addition, the NaCl-induced increases in c-caspase3, c-caspase8, and c-PRAP were reversed by treatment with Ala. These results indicate that Ala alleviates renal dysfunction via attenuating the apoptosis of the kidneys induced by salt loading.

The most frequent forms of ROS include superoxide anions, hydroxyl radicals, hypochlorous acid, hydrogen peroxide, singlet oxygen, and lipid peroxides, which are involved in various biological processes, such as cell growth, differentiation, development, and death [27]. Importantly, oxidative stress is implicated in the development and progression of kidney disease [28, 29]. Notably, Ala ameliorates oxidative stress in various cardiovascular diseases [10, 11, 20], but this phenomenon has not been well explored in renal diseases. In this study, we found that the decreases in the SOD activity and GSH level, and the increases in MDA, superoxide anions, and 8-OHdG induced by NaCl in the kidney, were reversed by Ala treatment. The increases in Rac1, p22^phox^, p47^phox^, and NOX4 induced by NaCl in the HK-2 cells were inhibited by Ala. In addition, DETC, an inhibitor of SOD, reversed the suppressive effects of Ala on the rise in the levels of c-caspase3, c-caspase8, and c-PARP induced by NaCl in the HK-2 cells. Our results show that Ala ameliorates NaCl-induced apoptosis of the renal tubular cells *via* oxidative-stress attenuation.

Several signaling pathways are involved in renal cell diseases [30, 31], such as PKC [32]. Ala alleviated cardiomyocyte hypertrophy, cardiac fibrosis, and artery remodeling *via* inhibiting the PKA [9], Akt [33], and p38MAPK [34] signaling pathways, respectively. Here, we aimed to explore the downstream signaling pathway involved in the effects of Ala on the HK-2 cell apoptosis. Our results showed that the PKC level was elevated in NaCl-treated HK-2 cells, but this increase was inhibited by Ala administration. PKC overexpression reversed the attenuating influence of Ala on the NaCl-induced increase in c-caspase3, c-caspase8, and c-PRAP in the HK-2 cells. These findings show that Ala alleviates the apoptosis of renal tubular cells *via* inhibition of the PKC signaling pathway. In addition to PKC pathway, many other pathways were involved in renal apoptosis [35–37]. Further signaling pathways will be explored in our next studies.

In conclusion, high-salt diets cause hypertension, which could be remedied by Ala. Salt loading induced kidney damage, but this damage was alleviated by Ala. Specifically, Ala alleviated renal tubular cells’ apoptosis *via* oxidative stress attenuation and PKC signaling pathway suppression. Therefore, Ala can be used for the therapy of renal disease-associated hypertension in the future.

## Data Availability

The data that were analyzed during the current study are available from the corresponding author on reasonable request.
